# Reduced 24-hour heart rate variability in neurosyphilis: a retrospective case–control study

**DOI:** 10.3389/fneur.2026.1935888

**Published:** 2026-09-17

**Authors:** Shouyue Jin, Jingjing She, Yanan Zhao, Weiwei Gao, Sicong Ma, Qingwei Yang, Xingyu Chen

**Affiliations:** 1 Department of Neurology, Zhongshan Hospital of Xiamen University, School of Medicine, Xiamen University, Xiamen, China; 2 The School of Clinical Medicine, Fujian Medical University, Fuzhou, Fujian, China; 3 Department of Neurology, The First Affiliated Hospital of Hunan University of Medicine, Huaihua, China

**Keywords:** cardiac autonomic regulation, daytime-to-nighttime changes, heart rate variability, Holter monitoring, neurosyphilis

## Abstract

**Background:**

Neurosyphilis may involve central or peripheral autonomic pathways, but its association with cardiac autonomic regulation has not been systematically evaluated. We compared 24-h heart rate variability (HRV) between patients with neurosyphilis and controls without known neurologic disease and examined daytime-to-nighttime changes and associated clinical factors.

**Methods:**

This single-center retrospective case–control study included 98 patients with neurosyphilis and 53 controls, all of whom underwent 24-h Holter monitoring. The primary endpoint was the standard deviation of all normal-to-normal intervals over 24 h (SDNN). Log-transformed HRV measures were analyzed using multivariable linear regression adjusted for demographic characteristics, vascular and metabolic risk factors, smoking status, and renal function. Multiple comparisons across secondary endpoints were controlled using the Benjamini–Hochberg procedure. Linear mixed-effects models evaluated daytime-to-nighttime changes, and exploratory analyses examined neurologic involvement and disability in patients with neurosyphilis.

**Results:**

Median 24-h SDNN was 112.5 ms in the neurosyphilis group and 128.0 ms in controls. After multivariable adjustment, SDNN was 15.3% lower in the neurosyphilis group [geometric mean ratio (GMR), 0.847; 95% confidence interval (CI), 0.773–0.928; *p* < 0.001]. Five of seven secondary time and frequency-domain endpoints were also lower after correction for multiple comparisons, whereas the ratio of low-frequency to high-frequency power did not differ significantly between groups. The nighttime increase in the root mean square of successive differences (RMSSD) was smaller in the neurosyphilis group (group-by-period interaction GMR, 0.854; 95% CI, 0.747–0.975; false discovery rate–adjusted *p* = 0.030). Among patients with neurosyphilis, each 1-point higher admission modified Rankin Scale score was associated with an 8.2% lower SDNN (GMR, 0.918; 95% CI, 0.881–0.956; *p* < 0.001). The primary estimate was similar after additional adjustment for mean heart rate and sleep quality, inverse probability of treatment weighting, and exclusion of participants with diabetes or patients with major neurologic involvement.

**Conclusion:**

Neurosyphilis was associated with lower values across several time and frequency-domain measures of 24-h HRV. Lower RMSSD and high-frequency power and a smaller nighttime increase in RMSSD were consistent with attenuation of vagally mediated short-term HRV.

## 1 Introduction

Syphilis remains a major global public health concern. In 2022, an estimated 8.0 million new cases of syphilis occurred among adults aged 15–49 years worldwide, up from 7.1 million in 2020. The World Health Organization’s target for 2030 is 710,000 new cases annually, corresponding to a 90% reduction from the 2020 baseline (1). *Treponema pallidum* can invade the central nervous system (CNS) early in the course of infection, and CNS involvement has been reported in approximately 30% of patients with early syphilis (2, 3). Neurosyphilis has a broad clinical spectrum, ranging from cerebrospinal fluid (CSF) abnormalities without overt neurologic symptoms to meningeal, cerebrovascular, or parenchymal disease. Clinical manifestations include cognitive decline, psychiatric and behavioral changes, and focal neurologic deficits (2, 4). Because no single test can definitively establish or exclude the diagnosis, neurosyphilis poses a persistent diagnostic challenge (3, 5).

Evidence that neurosyphilis affects cardiac autonomic regulation is limited. Early studies identified impaired baroreflex afferent function in patients with tabes dorsalis and orthostatic hypotension (6). Patients with tabes dorsalis also had markedly attenuated ventilatory responses to hypoxia, suggesting impaired carotid chemoreceptor afferent function (7). More recently, paroxysmal sympathetic hyperactivity was reported in a single patient with neurosyphilis (8). These reports raise the possibility that syphilis-related neurologic disease involves autonomic pathways. However, current evidence comes mainly from case reports and small case series, and quantitative studies using ambulatory electrocardiographic monitoring are lacking.

Heart rate variability (HRV) can be derived from ambulatory electrocardiographic recordings and is widely used as a noninvasive measure of cardiac autonomic regulation (9). The standard deviation of all normal-to-normal (NN) intervals over 24 h (SDNN) reflects overall HRV. The root mean square of successive differences between NN intervals (RMSSD) and high-frequency (HF) power predominantly reflect vagally mediated short-term HRV. Because HRV is also influenced by underlying heart rate, physical activity, posture, respiration, sleep, and recording period, no single measure can identify selective impairment of a particular autonomic branch. Lower HRV has been associated with adverse outcomes in several clinical populations (10–12). HRV is also used to investigate brain–heart autonomic regulation (13). Studies of type 2 diabetes, human immunodeficiency virus (HIV) infection, and Parkinson disease have reported abnormal HRV, although the patterns differ across disorders (14–16). Combined assessment of multiple HRV measures may better characterize overall alterations in cardiac autonomic regulation.

It is unknown whether patients with neurosyphilis have lower 24-h HRV and whether HRV is associated with neurologic manifestations or functional disability. We compared 24-h HRV between patients with neurosyphilis and controls without known neurologic disease. The primary endpoint was 24-h SDNN, and additional time and frequency-domain measures were evaluated as secondary endpoints. In exploratory analyses, we compared daytime-to-nighttime changes in HRV between groups and examined the associations of SDNN with clinical subtype, major neurologic involvement, and the modified Rankin Scale (mRS) score at admission.

## 2 Materials and methods

### 2.1 Study design and participants

This single-center retrospective case–control study included consecutive patients with neurosyphilis admitted to the Department of Neurology at Zhongshan Hospital, Xiamen University, from January 1, 2015, to May 1, 2026. Contemporaneous controls were identified from the Health Management Center of the same hospital. During routine health examinations, all controls underwent 24-h ambulatory electrocardiographic monitoring and brain magnetic resonance imaging (MRI). They had no known neurologic disease, and their Holter recordings showed no arrhythmias, marked ST–T changes, or other clinically significant abnormalities (Figure 1).

**Figure 1:**
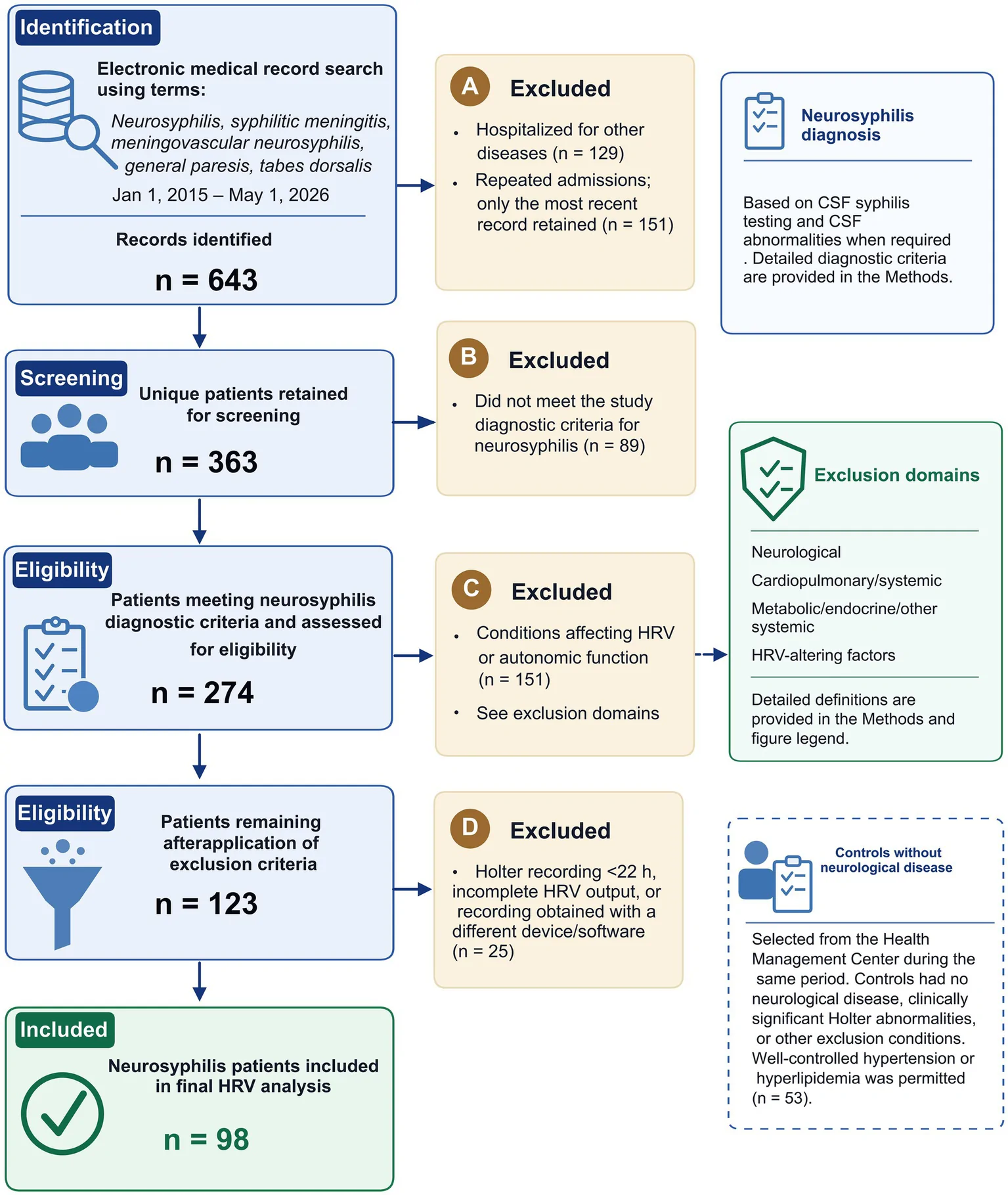
Selection of the study population. The study included 98 patients with neurosyphilis and 53 controls without a diagnosed neurological disorder. All participants had at least 22 h of valid ambulatory electrocardiographic recording available for heart rate variability analysis. MRI denotes Magnetic Resonance Imaging.

Neurosyphilis was diagnosed when both the CSF *Treponema pallidum* particle agglutination assay and the toluidine red unheated serum test were reactive. If only one of the two CSF syphilis tests was reactive, diagnosis additionally required a concomitant CSF abnormality, defined as a white blood cell count of ≥5 cells/μL or a protein concentration of >500 mg/L.

Participants in both groups were excluded if they had conditions known to affect HRV or cardiac autonomic regulation, including acute cerebrovascular disease, traumatic brain injury, moderate-to-severe, carotid stenosis, intracranial infection, epilepsy, ischemic heart disease, heart failure, atrial fibrillation, malignancy, an acute exacerbation of chronic obstructive pulmonary disease, obstructive sleep apnea, severe or poorly controlled hypertension or diabetes, HIV seropositivity, or use of β-blockers or atropine. Relevant medical histories were ascertained from inpatient records or health examination data, and neurologic lesions were assessed on the basis of MRI reports. Ischemic stroke, intracranial infection, and epilepsy attributable to syphilis were considered clinical manifestations of neurosyphilis and therefore did not constitute exclusion criteria for the neurosyphilis group.

The study was approved by the Ethics Committee of Zhongshan Hospital, Xiamen University (approval no. xmzsyyky2022-143), and was conducted in accordance with the Declaration of Helsinki. The requirement for informed consent was waived by the ethics committee because of the retrospective study design.

### 2.2 Clinical, laboratory, and MRI data

Age, sex, smoking status, vascular and metabolic risk factors, sleep quality, renal function, neurosyphilis-related clinical characteristics, laboratory findings, and MRI data were extracted from the electronic medical records. Vascular and metabolic risk factors included hypertension, diabetes, hyperlipidemia, and hyperuricemia. Participants were classified as ever or never smokers. Sleep quality was classified as normal or poor according to the medical records. Renal function was categorized into five groups according to the estimated glomerular filtration rate (eGFR): G1, eGFR ≥90 mL/min/1.73 m^2^; G2, 60–89 mL/min/1.73 m^2^; G3, 30–59 mL/min/1.73 m^2^; G4, 15–29 mL/min/1.73 m^2^; and G5, <15 mL/min/1.73 m^2^.

Neurologic disability was assessed using the mRS at admission. On the basis of clinical manifestations and MRI findings, neurosyphilis was classified into asymptomatic, meningovascular-spectrum, and parenchymal forms. The meningovascular spectrum comprised meningovascular neurosyphilis, syphilitic meningitis, and meningoencephalitis. Parenchymal neurosyphilis comprised general paresis, tabes dorsalis, and cerebral gumma.

Major neurologic involvement was defined, on the basis of clinical and MRI findings, as syphilis-related ischemic stroke, intracerebral hemorrhage, cerebral venous sinus thrombosis, meningoencephalitis, hydrocephalus, cerebral gumma, or a documented seizure. All participants underwent brain MRI. Mild intracranial atherosclerotic changes, cerebral atrophy, and Fazekas grade 1 or 2 white matter hyperintensities were permitted in the control group; controls with any other definite neurologic lesion were excluded. Laboratory data for patients with neurosyphilis included serum and CSF syphilis test results, CSF white blood cell counts, and CSF protein concentrations.

### 2.3 Holter monitoring and HRV assessment

All participants underwent 24-h ambulatory electrocardiographic (ECG) monitoring. Monitoring in patients with neurosyphilis began on the second day after admission, which was the first day of antibiotic treatment. Controls were fitted with the recording device at the Health Management Center and completed monitoring during their usual daily activities. Participants were instructed to maintain their usual routines as far as possible and to avoid strenuous exercise, alcohol consumption, and excessive caffeine intake.

ECG data were acquired using a DMS 300-4A recorder and analyzed with CardioScan Premier DM ambulatory ECG analysis software (DM Software Inc., Beijing, China). The ECG signal was sampled at 128 Hz, corresponding to a nominal sampling interval of approximately 7.8 ms. Only recordings containing at least 22 h of valid data were included.

The CardioScan software uses proprietary algorithms for R-wave detection, beat classification, and identification of artifacts and ectopic beats. Beat-by-beat data were classified as normal sinus beats, ventricular ectopic beats, atrial ectopic beats, or artifacts. The software generated the NN interval series after excluding beats that did not meet its criteria. No manual correction of *R*-wave positions or reclassification of beat types and artifact labels was performed. Recordings with persistent nonsinus rhythm, prolonged signal loss, or an NN interval series that could not be generated reliably by the software were excluded from the analysis.

The daytime window was defined as 06:00–22:00 and the nighttime window as 22:00–06:00 the following day. The 24-h time-domain measures were SDNN, the standard deviation of the mean NN intervals across all 5-min segments (SDANN), RMSSD, and the percentage of successive NN interval differences exceeding 50 ms (pNN50). Daytime and nighttime SDNN, RMSSD, and pNN50 were also extracted, together with the mean 24-h heart rate. A pNN50 value of zero indicated that the software detected no successive NN intervals differing by more than 50 ms in the valid NN interval series. Because the corrected beat-by-beat NN interval series and the parameters governing interval quantization were not retained, zero pNN50 values could not be independently recalculated at the beat level.

Frequency-domain analysis was performed using CardioScan’s proprietary HRV module. The software applied fast Fourier transformation to consecutive 5-min segments. The low-frequency (LF) band was defined as 0.040–0.150 Hz and the HF band as 0.150–0.400 Hz. The software-generated 24-h summaries of total power, LF power, HF power, and the LF/HF ratio were extracted.

Because CardioScan is proprietary software, the available technical documentation did not specify the thresholds used for R-wave detection or the criteria used to identify artifacts and ectopic beats. It also did not provide the detailed parameters used for interpolation or correction of excluded NN intervals, uniform resampling, detrending, windowing, assessment of segment stationarity, handling of missing NN intervals, or aggregation of segment-level frequency-domain estimates.

### 2.4 Statistical analysis

Continuous variables are presented as mean (standard deviation) or median (interquartile range), according to their distributions, and categorical variables as number (percentage). The pre-specified primary endpoint was 24-h SDNN. Secondary endpoints included SDANN, RMSSD, pNN50, total power, LF power, HF power, and the LF/HF ratio. The daytime-to-nighttime HRV analyses and analyses restricted to patients with neurosyphilis were exploratory.

HRV measures were transformed using natural logarithms before analysis. For pNN50, which included zero values, ln (x + 1) transformation was used. Except for pNN50, model estimates were back-transformed and reported as geometric mean ratios (GMRs) comparing the neurosyphilis group with the control group, with 95% confidence intervals (CIs). For pNN50, the back-transformed estimates represented GMRs on the pNN50 + 1 scale, and the corresponding effects were interpreted on that scale.

The primary analysis used multivariable linear regression adjusted for age, sex, hypertension, diabetes, hyperlipidemia, hyperuricemia, smoking status, and eGFR category. HC3 heteroskedasticity-consistent standard errors were used. Additional models added mean 24-h heart rate or sleep quality individually, followed by a model that included both variables, to assess the robustness of the primary findings. The primary model was also repeated after participants with diabetes were excluded. The Benjamini–Hochberg procedure was used to control the false discovery rate (FDR) across the secondary HRV endpoints. The between-group difference in the frequency of zero pNN50 values was examined in an exploratory analysis using Fisher’s exact test.

Daytime and nighttime SDNN, RMSSD, and pNN50 were analyzed using linear mixed-effects models. Fixed effects included group, recording period, and the group-by-period interaction. A participant-specific random intercept was included, and the models were adjusted for the baseline covariates included in the primary analysis. The Benjamini–Hochberg procedure was applied to the three group-by-period interaction tests.

In a clinical sensitivity analysis, the primary model was repeated after patients with neurosyphilis and major neurologic involvement were excluded. A methodological sensitivity analysis was conducted using inverse probability of treatment weighting (IPTW). Propensity scores were estimated from the baseline covariates included in the primary model. Stabilized weights for the average treatment effect were calculated and truncated at the 1st and 99th percentiles. Covariate balance was assessed using standardized mean differences (SMDs), with an absolute SMD of <0.10 indicating acceptable balance. The weight distribution and effective sample size were also reported.

An IPTW-only weighted model and a doubly robust model that additionally adjusted for the same baseline covariates after weighting were fitted separately. Standard errors were calculated using robust estimators appropriate for weighted data. Group-stratified bootstrap resampling was performed with 5,000 replicates, with the propensity scores and weights re-estimated in each resample. Percentile and bias-corrected and accelerated (BCa) 95% CIs were reported for the bootstrap analyses. In the restricted sample excluding patients with major neurologic involvement, propensity scores and weights were re-estimated, and the IPTW-only and doubly robust models were repeated.

Among patients with neurosyphilis, associations of 24-h SDNN with disease subtype and admission mRS score were examined in separate multivariable linear regression models adjusted for the baseline covariates included in the primary analysis. The mRS score was entered into the model as a continuous variable, and the GMR was reported per 1-point increase. Overall differences among disease subtypes were assessed using an HC3 robust Wald test, with Tukey adjustment for pairwise comparisons.

For the analysis of major neurologic involvement, participants were classified into three groups: controls, patients with neurosyphilis without major neurologic involvement, and patients with neurosyphilis with major neurologic involvement. The overall difference among the three groups was assessed using an HC3 robust Wald test, and pairwise comparisons were adjusted using the Holm procedure.

E-values were calculated only for the primary endpoint (17). The group coefficient from the log-transformed SDNN model, its HC3 robust standard error, and the model residual standard deviation were entered into the EValue::evalues. OLS function. This method first standardizes the linear regression effect, approximates its conversion to the risk-ratio scale, and then calculates the E-value. E-values were reported for the point estimate and for the CI limit closest to the null. Because this calculation treats the model residual standard deviation as known, the E-value was regarded as a supplementary sensitivity measure for assessing unmeasured confounding.

All tests were two-sided, and *p* < 0.05 was considered statistically significant. Benjamini–Hochberg-adjusted *p* values were also reported for the secondary endpoints. All statistical analyses were performed using R version 4.6.0.

## 3 Results

### 3.1 Demographic and clinical characteristics

The study included 151 participants: 98 patients with neurosyphilis and 53 controls without known neurologic disease (Figure 1). Demographic and clinical characteristics are summarized in Table 1 and Supplementary Table S1. The mean age was 55.2 years in the neurosyphilis group and 56.1 years in the control group. Men accounted for 70.4 and 47.2% of the two groups, respectively. The corresponding proportions of ever smokers were 44.9 and 26.4%, and those with poor sleep quality were 44.9 and 26.4%. Hyperlipidemia was present in 44.9% of patients with neurosyphilis and 69.8% of controls, whereas hyperuricemia was present in 8.2 and 22.6%, respectively. Hypertension and diabetes were similarly prevalent in the two groups. The proportions classified as eGFR G2 were 12.2% in the neurosyphilis group and 7.5% in the control group. Mean 24-h heart rates were 72.2 and 70.6 beats/min, respectively.

**Table 1:** Baseline characteristics and unadjusted 24-h heart rate variability indices.

Characteristic	Overall (*N* = 151)	Controls without neurological disease (*n* = 53)	Neurosyphilis (*n* = 98)	*p*-value
Age, years	55.5 (12.0)	56.1 (10.8)	55.2 (12.6)	0.700
Male sex, *n* (%)	94 (62.3)	25 (47.2)	69 (70.4)	0.008
Hypertension, *n* (%)	58 (38.4)	20 (37.7)	38 (38.8)	>0.90
Hyperlipidemia, *n* (%)	81 (53.6)	37 (69.8)	44 (44.9)	0.004
Hyperuricemia, *n* (%)	20 (13.2)	12 (22.6)	8 (8.2)	0.021
Diabetes, *n* (%)	36 (23.8)	10 (18.9)	26 (26.5)	0.300
SDNN, ms	123.3 (33.2)	135.2 (32.7)	116.8 (31.7)	0.001
SDANN, ms	107.7 (31.9)	118.4 (32.8)	101.9 (30.0)	0.003
RMSSD, ms	39.0 (27.5)	47.1 (32.9)	34.7 (23.1)	0.017
pNN50, %	7.7 (9.3)	9.5 (9.0)	6.8 (9.4)	0.083
Total power, ms^2^	2,286.5 (1,322.5)	2,447.9 (1,212.7)	2,199.2 (1,376.4)	0.300
LF power, ms^2^	423.7 (304.5)	477.0 (320.0)	394.9 (293.4)	0.120
HF power, ms^2^	181.2 (158.9)	220.4 (157.5)	160.0 (156.3)	0.026
LF/HF ratio	3.1 (2.0)	2.5 (1.4)	3.5 (2.2)	0.001

Values are mean (SD) unless otherwise indicated. *p* values are from the unadjusted between-group comparisons reported in the source table. HF, high frequency; HRV, heart rate variability; LF, low frequency; pNN50, percentage of adjacent NN intervals differing by >50 ms; RMSSD, root mean square of successive NN-interval differences; SDANN, standard deviation of the averages of NN intervals in all 5-min segments; SDNN, standard deviation of all NN intervals.

### 3.2 Primary endpoint: 24-hour SDNN

The median 24-h SDNN was 112.5 ms (interquartile range, 98.0–133.5) in the neurosyphilis group and 128.0 ms (interquartile range, 115.0–148.0) in the control group (Figure 2A; Supplementary Table S2). After adjustment for age, sex, hypertension, diabetes, hyperlipidemia, hyperuricemia, smoking status, and eGFR category, SDNN was lower in the neurosyphilis group than in the control group (GMR, 0.847; 95% CI, 0.773–0.928; *p* < 0.001), corresponding to a 15.3% lower adjusted geometric mean (Supplementary Table S3).

**Figure 2:**
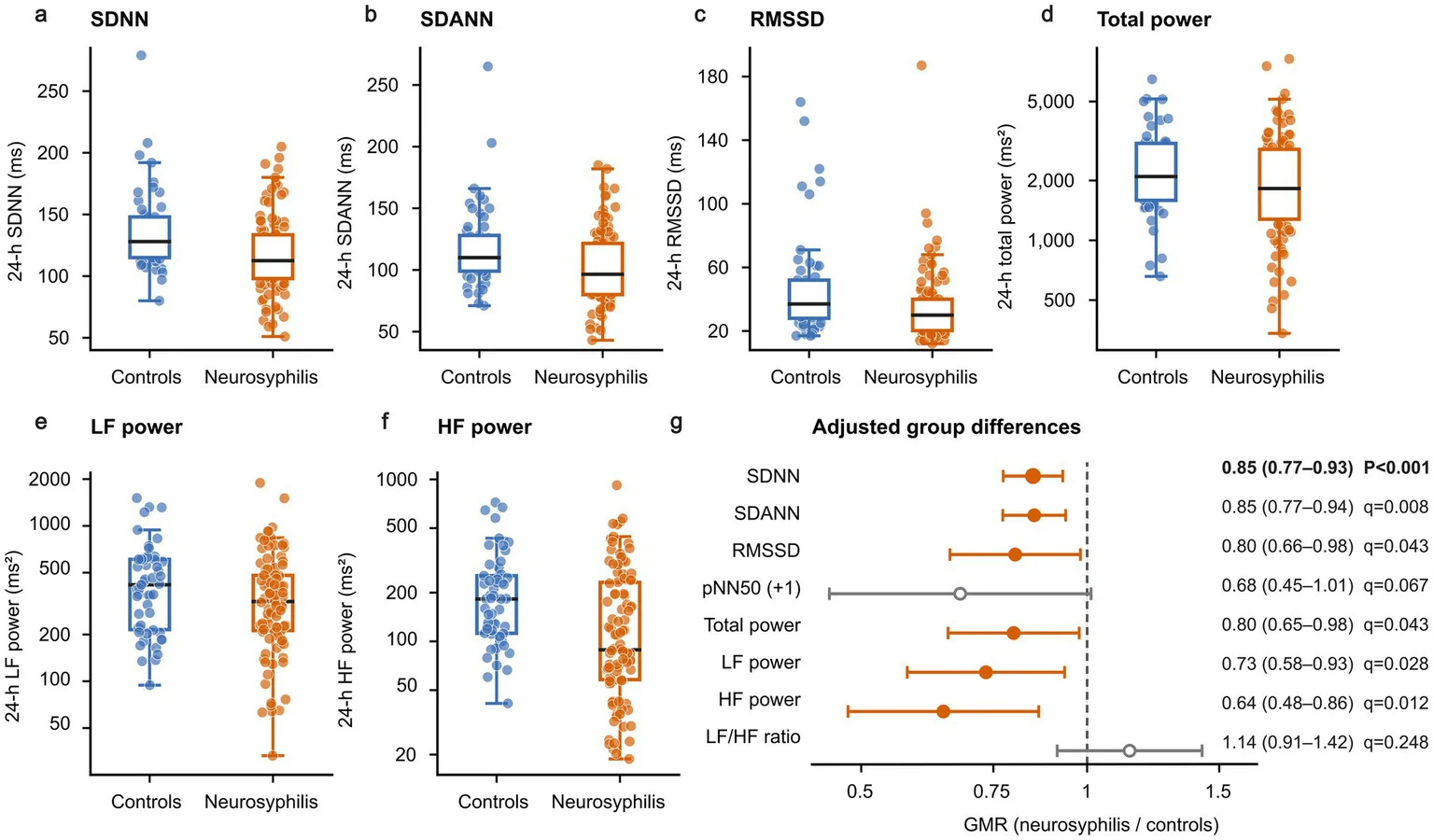
Twenty-four-hour heart rate variability in patients with neurosyphilis and controls. **(a–f)** Distributions of SDNN, SDANN, RMSSD, total power, LF power, and HF power, respectively. **(g)** Adjusted geometric mean ratios (GMRs) and 95% confidence intervals comparing patients with controls. Models were adjusted for age, sex, hypertension, diabetes, hyperlipidemia, hyperuricemia, smoking history, and estimated glomerular filtration rate category. GMRs below 1 indicate lower values in patients. The pNN50 estimate is expressed on the pNN50 + 1 scale. The primary endpoint, SDNN, was not adjusted for multiplicity; *q* values denote Benjamini–Hochberg-adjusted *p* values for the seven secondary endpoints. SDNN, standard deviation of all normal-to-normal intervals; SDANN, standard deviation of mean normal-to-normal intervals across 5-min segments; RMSSD, root mean square of successive normal-to-normal interval differences; pNN50, percentage of successive normal-to-normal intervals differing by >50 ms; LF, low frequency; HF, high frequency.

### 3.3 Secondary HRV endpoints

Five of the seven secondary HRV endpoints remained statistically significant after FDR correction. Compared with controls, patients with neurosyphilis had lower SDANN (GMR, 0.850; 95% CI, 0.772–0.936; FDR-adjusted *p* = 0.008), RMSSD (GMR, 0.802; 95% CI, 0.657–0.980; FDR-adjusted *p* = 0.043), and total power (GMR, 0.798; 95% CI, 0.653–0.976; FDR-adjusted *p* = 0.043). LF power (GMR, 0.733; 95% CI, 0.576–0.933; FDR-adjusted *p* = 0.028) and HF power (GMR, 0.643; 95% CI, 0.480–0.862; FDR-adjusted *p* = 0.012) were also lower in the neurosyphilis group (Figures 2B–G).

After ln (pNN50 + 1) transformation, the between-group difference on the pNN50 + 1 scale was not statistically significant after FDR correction (GMR, 0.678; 95% CI, 0.453–1.012; FDR-adjusted *p* = 0.067). The LF/HF ratio also did not differ significantly between groups (GMR, 1.139; 95% CI, 0.912–1.423; FDR-adjusted *p* = 0.248).

In a separate exploratory analysis, 18 patients with neurosyphilis (18.4%) and 2 controls (3.8%) had a 24-h pNN50 value of zero. Zero pNN50 values were more frequent in the neurosyphilis group according to Fisher’s exact test (odds ratio, 5.69; 95% CI, 1.27–52.60; *p* = 0.011; Supplementary Table S2).

### 3.4 Daytime and nighttime HRV patterns

Adjusted linear mixed-effects models showed between-group differences in the daytime-to-nighttime changes in RMSSD and pNN50 + 1. The group-by-period interaction, expressed as the ratio of the nighttime-to-daytime GMR in the neurosyphilis group to that in the control group, was 0.854 for RMSSD (95% CI, 0.747–0.975; FDR-adjusted *p* = 0.030) and 0.684 on the pNN50 + 1 scale (95% CI, 0.526–0.890; FDR-adjusted *p* = 0.015). The group-by-period interaction for SDNN was not statistically significant (ratio, 0.989; 95% CI, 0.910–1.074; FDR-adjusted *p* = 0.787; Figure 3; Supplementary Table S4).

**Figure 3:**
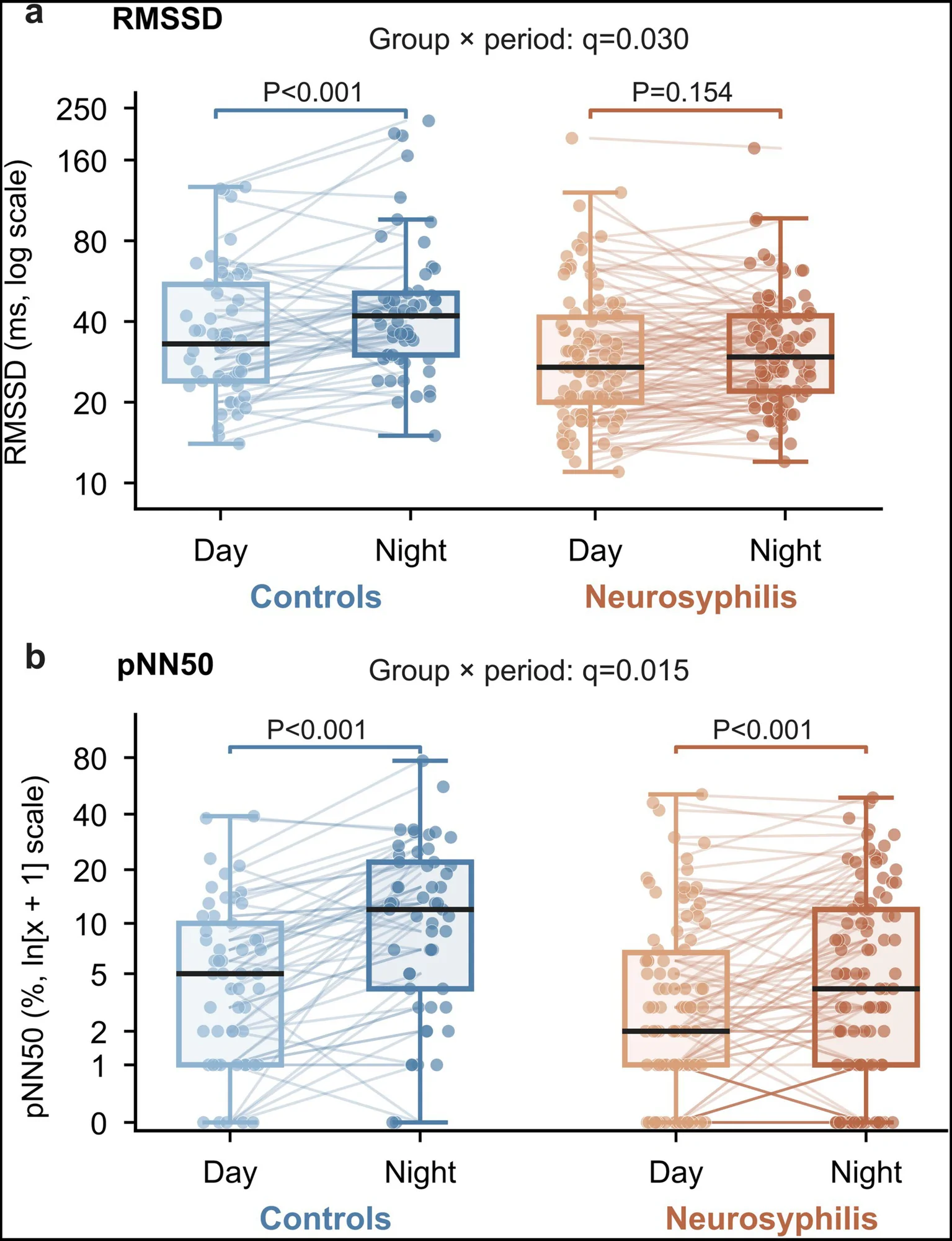
Daytime and nighttime heart rate variability in patients with neurosyphilis and controls. **(a)** RMSSD and **(b)** pNN50; lines connect measurements from the same participant. Daytime was defined as 06:00–22:00 and nighttime as 22:00–06:00. Comparisons used linear mixed-effects models with group, period, and their interaction as fixed effects and a participant-specific random intercept, adjusted for age, sex, hypertension, diabetes, hyperlipidemia, hyperuricemia, smoking history, and estimated glomerular filtration rate category. Within-group *p* values compare nighttime with daytime; *q* values denote group-by-period interaction p values adjusted across SDNN, RMSSD, and pNN50 using the Benjamini–Hochberg procedure. pNN50 estimates are expressed on the pNN50 + 1 scale. Nighttime intervals do not represent confirmed sleep. RMSSD, root mean square of successive normal-to-normal interval differences; pNN50, percentage of successive normal-to-normal intervals differing by >50 ms; SDNN, standard deviation of all normal-to-normal intervals.

In the control group, nighttime RMSSD was higher than daytime RMSSD (nighttime-to-daytime GMR, 1.240; 95% CI, 1.114–1.380; *p* < 0.001). The daytime-to-nighttime difference was not statistically significant in the neurosyphilis group (nighttime-to-daytime GMR, 1.059; 95% CI, 0.979–1.146; *p* = 0.154). On the pNN50 + 1 scale, the nighttime geometric mean was higher than the daytime geometric mean in both groups, but the relative increase was smaller in the neurosyphilis group. The nighttime-to-daytime GMR was 1.965 (95% CI, 1.591–2.428) in the control group and 1.345 (95% CI, 1.151–1.571) in the neurosyphilis group.

### 3.5 Exploratory analyses of neurologic involvement, disease subtype, and disability

Among the 98 patients with neurosyphilis, 35 (35.7%) had major neurologic involvement and 63 (64.3%) did not. Adjusted SDNN differed across controls, patients with neurosyphilis without major neurologic involvement, and patients with neurosyphilis with major neurologic involvement (HC3 robust Wald test, *p* < 0.001; Figure 4A; Supplementary Table S5).

**Figure 4:**
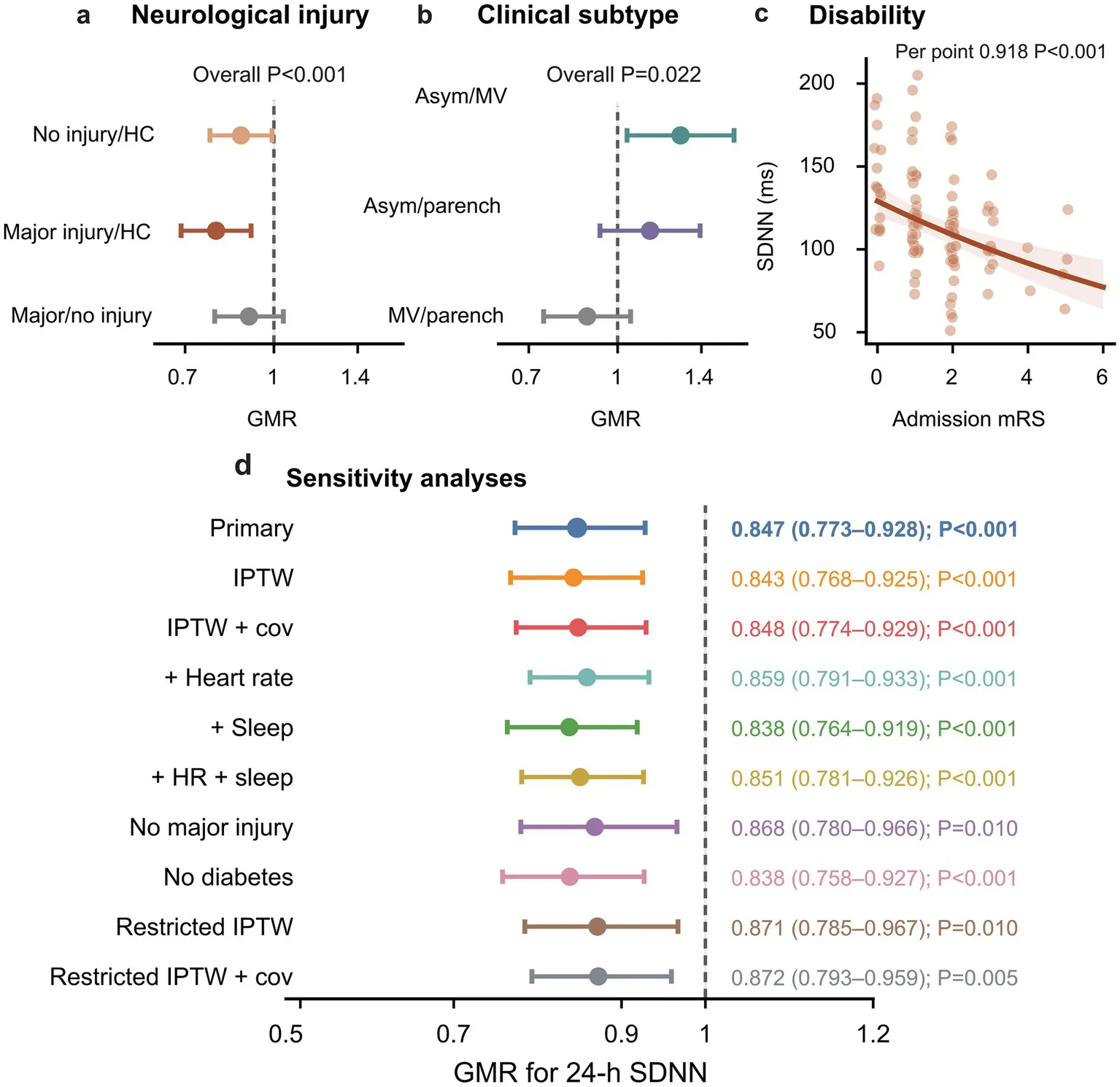
Clinical associations and sensitivity analyses of 24-hour SDNN. **(a)** Comparisons among controls and patients with or without major neurological involvement. **(b)** Comparisons across neurosyphilis subtypes. **(c)** Association with admission modified Rankin Scale (mRS) score; the geometric mean ratio (GMR) is reported per 1-point increase. Models in **(a–c)** were adjusted for age, sex, hypertension, diabetes, hyperlipidemia, hyperuricemia, smoking history, and estimated glomerular filtration rate category. Overall p values in **(a)** and **(b)** were obtained using HC3 robust Wald tests, with Holm- and Tukey-adjusted pairwise comparisons, respectively. (d) Sensitivity analyses using weighting, additional adjustment for mean heart rate and/or sleep quality, and exclusions based on major neurological involvement or diabetes. Restricted weighted analyses excluded patients with major neurological involvement and re-estimated propensity scores and weights. Points and horizontal bars in **(a)**, **(b)**, and **(d)** indicate GMRs and 95% confidence intervals. HC, controls; Asym, asymptomatic; MV, meningovascular-spectrum; parench, parenchymal; IPTW, inverse probability of treatment weighting; cov, baseline covariates; HR, mean 24-hour heart rate; SDNN, standard deviation of all normal-to-normal intervals.

Compared with controls, SDNN was lower in patients with neurosyphilis without major neurologic involvement (GMR, 0.876; 95% CI, 0.773–0.993; Holm-adjusted *p* = 0.023) and in those with major neurologic involvement (GMR, 0.793; 95% CI, 0.689–0.912; Holm-adjusted *p* < 0.001). Among patients with neurosyphilis, SDNN did not differ significantly between those with and without major neurologic involvement (GMR for patients with vs. without major neurologic involvement, 0.905; 95% CI, 0.788–1.039; Holm-adjusted *p* = 0.081).

Of the patients with neurosyphilis, 15 had asymptomatic neurosyphilis, 41 had meningovascular-spectrum disease, and 42 had parenchymal disease. Adjusted SDNN differed across the three clinical subtypes (HC3 robust Wald test, *p* = 0.022). SDNN was higher in patients with asymptomatic neurosyphilis than in those with meningovascular-spectrum disease (GMR, 1.287; 95% CI, 1.038–1.596; Tukey-adjusted *p* = 0.017). No statistically significant differences were found between the asymptomatic and parenchymal groups (GMR, 1.139; 95% CI, 0.931–1.393; Tukey-adjusted *p* = 0.276) or between the meningovascular-spectrum and parenchymal groups (GMR, 0.885; 95% CI, 0.743–1.053; Tukey-adjusted *p* = 0.221; Figure 4B).

After adjustment for the same baseline covariates, each 1-point increase in the admission mRS score was associated with an 8.2% lower adjusted geometric mean of SDNN among patients with neurosyphilis (GMR, 0.918; 95% CI, 0.881–0.956; *p* < 0.001; Figure 4C).

### 3.6 Sensitivity analyses

IPTW improved the balance of most baseline covariates, although mild residual imbalance remained for hypertension and smoking status, with absolute SMDs of 0.122 and 0.104, respectively. After truncation at the 1st and 99th percentiles, the weights ranged from 0.418 to 2.419. The overall effective sample size was 127.0, with group-specific effective sample sizes of 86.4 for the neurosyphilis group and 40.8 for the control group.

The primary association was similar across model specifications. In the IPTW-only weighted model, the GMR for SDNN was 0.843 (95% CI, 0.768–0.925; *p* < 0.001). In the doubly robust model with additional covariate adjustment after weighting, the GMR was 0.848 (95% CI, 0.774–0.929; *p* < 0.001). All 5,000 group-stratified bootstrap resamples yielded valid estimates. The BCa bootstrap 95% CI for the IPTW estimate was 0.767–0.923 (Figure 4D; Supplementary Table S6).

Additional adjustment for mean 24-h heart rate did not materially alter the primary result (GMR, 0.859; 95% CI, 0.791–0.933; *p* < 0.001). Similar estimates were obtained after additional adjustment for sleep quality (GMR, 0.838; 95% CI, 0.764–0.919; *p* < 0.001) and after simultaneous adjustment for mean heart rate and sleep quality (GMR, 0.851; 95% CI, 0.781–0.926; *p* < 0.001). After participants with diabetes were excluded, neurosyphilis remained associated with lower SDNN (*n* = 115; GMR, 0.838; 95% CI, 0.758–0.927; *p* < 0.001).

After the 35 patients with major neurologic involvement were excluded, the restricted sample comprised 63 patients with neurosyphilis and 53 controls. In the refitted primary model, SDNN remained lower in the neurosyphilis group (GMR, 0.868; 95% CI, 0.780–0.966; *p* = 0.010). After the propensity scores and weights were re-estimated in this restricted sample, the absolute SMDs for all modeled covariates were below 0.10. Similar estimates were obtained from the IPTW-only weighted model (GMR, 0.871; 95% CI, 0.785–0.967; *p* = 0.010) and the doubly robust model with additional covariate adjustment after weighting (GMR, 0.872; 95% CI, 0.793–0.959; *p* = 0.005).

The *E*-value for the primary SDNN association was 3.06, and the *E*-value for the CI limit closest to the null was 1.96 (Supplementary Table S7).

## 4 Discussion

In this single-center retrospective case–control study of 98 patients with neurosyphilis and 53 controls, the adjusted geometric mean of 24-h SDNN was 15.3% lower in patients with neurosyphilis after adjustment for demographic characteristics, vascular and metabolic risk factors, smoking status, and renal function. SDANN, RMSSD, total power, LF power, and HF power were also lower. The adjusted geometric means of HF power and RMSSD were 35.7 and 19.8% lower, respectively, whereas the LF/HF ratio did not differ significantly between groups. Patients with neurosyphilis also had a smaller nighttime increase in RMSSD, with a similar between-group difference in daytime-to-nighttime changes on the pNN50 + 1 scale. The primary result was largely unchanged after further adjustment for mean heart rate and sleep quality, exclusion of participants with diabetes or patients with major neurologic involvement, and IPTW. These findings indicate that neurosyphilis is associated with reductions in several time and frequency-domain HRV measures, with particularly marked reductions in measures of vagally mediated short-term HRV.

Previous evidence of autonomic involvement in neurosyphilis has come mainly from case reports and small case series. Orthostatic hypotension, abnormal baroreflex afferent function, and attenuated hypoxic responses have been reported in patients with tabes dorsalis, and a more recent case report described paroxysmal sympathetic hyperactivity associated with neurosyphilis (6–8). These observations suggest that syphilis may affect central or peripheral autonomic pathways, but quantitative evidence based on long-term ambulatory ECG monitoring remains limited. By examining HRV measures derived from 24-h Holter recordings, the present study quantified both overall HRV and daytime-to-nighttime changes, extending evidence previously based largely on clinical manifestations and autonomic reflex testing.

Individual HRV measures reflect overlapping but distinct aspects of heart rate regulation (18). Twenty-four-hour SDNN represents overall variability across the recording period and is influenced by autonomic regulation, daytime-to-nighttime changes, sleep, physical activity, and posture. SDANN primarily captures slower fluctuations in mean NN intervals across 5-min segments, whereas total power quantifies overall spectral variability within the analyzed frequency range. RMSSD and pNN50 reflect short-term, beat-to-beat variation in NN intervals. HF power primarily corresponds to respiratory sinus arrhythmia and is generally regarded as an indirect measure of vagally mediated HRV (9, 18, 19). LF power is influenced by parasympathetic modulation, baroreflex function, and, to some extent, sympathetic mechanisms and therefore cannot be regarded as a pure measure of sympathetic activity. Similarly, the LF/HF ratio should not be interpreted as a direct measure of sympathovagal balance (20–22).

The concurrent reductions in SDNN, SDANN, and total power support the presence of lower overall HRV in patients with neurosyphilis. The relatively large reductions in RMSSD and HF power are consistent with attenuated vagally mediated short-term HRV. The absence of a significant between-group difference in the LF/HF ratio does not establish that autonomic balance was preserved, because proportional reductions in both LF and HF power may leave their ratio largely unchanged.

Lower HRV in neurosyphilis may reflect a combination of CNS involvement and nonspecific disease-related factors. According to the neurovisceral integration model, the prefrontal cortex, limbic structures, hypothalamus, and autonomic nuclei in the brainstem participate in cardiac autonomic regulation, and structural or functional abnormalities within this network have been associated with lower HRV (13). Neurosyphilis may affect these regions through meningovascular ischemia, parenchymal inflammation, and other CNS lesions (2, 23). Inflammation, infection-related stress, and sleep disturbance may also alter central autonomic outflow, baroreflex function, or sinoatrial node responsiveness.

The exploratory analyses could not distinguish among these mechanisms but provided two additional observations. First, adjusted SDNN was lower than in controls among patients with neurosyphilis both with and without syphilis-related stroke, epilepsy, meningoencephalitis, or other major neurologic involvement. SDNN did not differ significantly between patients with and without major neurologic involvement. After patients with major neurologic involvement were excluded, SDNN remained 13.2% lower than in controls, suggesting that these overt neurologic manifestations did not fully account for the observed association.

Second, each 1-point higher admission mRS score was associated with an 8.2% lower SDNN among patients with neurosyphilis, linking lower SDNN to greater concurrent neurologic disability. Because mRS and HRV were assessed during the same hospitalization, this association does not establish that HRV can track longitudinal changes in disability. Exploratory differences were also observed across clinical subtypes, but the asymptomatic subgroup included only 15 patients, limiting the precision of these estimates. Confirmation in larger samples is needed.

Concurrent reductions in multiple time and frequency-domain HRV measures are not specific to neurosyphilis. A meta-analysis of 24-h Holter studies in type 2 diabetes reported reductions in SDNN, RMSSD, pNN50, total power, LF power, and HF power without a clear change in the LF/HF ratio, a pattern similar to that observed in the present study (14). Studies of HIV infection, multiple sclerosis, and anti–N-methyl-D-aspartate receptor encephalitis have also reported various abnormalities in HRV or autonomic regulation (15, 24–26). However, the reported patterns of LF power, HF power, and the LF/HF ratio have differed among diseases. These differences may reflect variation in lesion location, inflammatory state, disease duration, and treatment status.

Infection-associated HRV abnormalities may also change over time. In a longitudinal study of health care workers with mild infection caused by severe acute respiratory syndrome coronavirus 2 (SARS-CoV-2), Liviero et al. found that HRV abnormalities detected early after infection persisted for a period but had largely resolved approximately 6 months after the first negative molecular test (27). Autonomic changes after SARS-CoV-2 infection are not directly comparable with those associated with chronic neurosyphilis. Nevertheless, these longitudinal findings illustrate that a single HRV assessment cannot distinguish a transient response to illness from persistent autonomic dysregulation. Whether HRV in neurosyphilis varies with disease stage or improves after effective antibiotic treatment requires prospective monitoring before and after treatment.

Daytime-to-nighttime HRV changes also differed between patients with neurosyphilis and controls. RMSSD increased by 24.0% from daytime to nighttime in controls but by only 5.9% in patients with neurosyphilis, with a statistically significant group-by-period interaction. A similar interaction was observed on the pNN50 + 1 scale. However, given the distribution of zero pNN50 values and the fact that the between-group difference in 24-h pNN50 was not statistically significant after FDR correction, this result should be regarded as exploratory.

Polysomnographic studies have shown that sympathetic activity generally decreases during sleep, particularly during non–rapid eye movement and slow-wave sleep, whereas vagally mediated HRV increases (28, 29). Attenuation of the normal parasympathetic increase during non–rapid eye movement sleep has also been reported in neurodegenerative diseases (30). These findings provide a physiologic context for the nighttime results in the present study. However, our analyses used fixed clock-time windows and did not establish actual sleep times or sleep stages. The smaller nighttime increase in RMSSD is therefore consistent with attenuation of the nighttime increase in vagally mediated short-term HRV but does not establish a sleep-stage-specific abnormality or disruption of endogenous circadian rhythms.

Several limitations should be considered. First, the single-center retrospective case–control design cannot establish a causal relationship between neurosyphilis and lower HRV and did not assess post-treatment changes in HRV or longitudinal clinical outcomes. Holter monitoring began on the first day of antibiotic treatment in all patients with neurosyphilis. The measurements therefore cannot be regarded as strictly pretreatment baselines, and an early treatment effect cannot be excluded. Controls were identified through the Health Management Center, but the specific indications for Holter monitoring were not fully preserved, creating the potential for selection bias. Patients underwent monitoring in the hospital, whereas controls completed monitoring during their usual daily activities. The groups may therefore have differed systematically in physical activity, posture, and opportunities for sleep. Although the models adjusted for several clinical factors and additionally accounted for mean heart rate and sleep quality, residual confounding by physical activity, respiratory patterns, psychological state, body mass index, and other unmeasured factors remains possible.

Second, HRV is an indirect measure of cardiac autonomic regulation. Respiration, continuous blood pressure, and conventional autonomic reflexes were not recorded concurrently. The lower HF power therefore cannot be attributed exclusively to attenuation of vagally mediated HRV, and the specific autonomic pathways involved cannot be determined. The fixed daytime and nighttime windows were not verified by polysomnography, actigraphy, or sleep diaries and cannot be equated with actual sleep–wake states, specific sleep stages, or endogenous circadian rhythms. The database recorded only that included recordings met the minimum validity threshold of 22 h; the distribution of actual recording durations was unavailable.

HRV estimation also depended on proprietary software algorithms. The software did not output or retain the proportion of ectopic or artifactual beats excluded or corrected in each recording. Signal quality therefore could not be assessed on the basis of correction rates, and corresponding sensitivity analyses could not be performed. The sampling rate of 128 Hz, the absence of retained beat-by-beat NN interval series, and the lack of documentation for some frequency-domain processing parameters may also limit the reproducibility of the pNN50 and frequency-domain findings. Finally, the asymptomatic neurosyphilis subgroup included only 15 patients. The subtype analyses and other exploratory findings require confirmation in larger, multicenter studies using standardized recording conditions.

## 5 Conclusion

Neurosyphilis was associated with lower values across several 24-h time and frequency-domain HRV measures. Reductions in measures of vagally mediated short-term HRV were particularly apparent, together with smaller nighttime increases in RMSSD and on the pNN50 + 1 scale. Standardized prospective multicenter studies are needed to clarify the underlying mechanisms, reversibility with treatment, and clinical significance of these findings.

## Data Availability

The original contributions presented in the study are included in the article/Supplementary material, further inquiries can be directed to the corresponding authors.

## References

[1] World Health Organization. Implementing the Global Health sector Strategies on HIV, Viral Hepatitis and Sexually Transmitted Infections, 2022–2030: 2024 progress Report. Geneva: World Health Organization (2024).

[2] Hamill MM Ghanem KG Tuddenham S. State-of-the-art review: neurosyphilis. Clin Infect Dis. (2024) 78:e57–68. doi: 10.1093/cid/ciad437 37593890

[3] Marra CM. Neurosyphilis. Brain. (2025) 148:4222–40. doi: 10.1093/brain/awaf329, 40911405

[4] Kaur B Khanna D. A narrative review of the many psychiatric manifestations of neurosyphilis: the great imitator. Cureus. (2023) 15:e44866. doi: 10.7759/cureus.44866, 37814742 PMC10560128

[5] Tuddenham S Ghanem KG. Neurosyphilis: knowledge gaps and controversies. Sex Transm Dis. (2018) 45:147–51. doi: 10.1097/OLQ.0000000000000723, 29420441 PMC5808573

[6] Tilbery CP Gagliardi R Sanvito WL Stella FP Ferlante LES. Hipotensão postural na tabes dorsalis. Registro de um caso [Postural hypotension in tabes dorsalis. Report of a case]. Arq Neuropsiquiatr. (1977) 35:73–9. doi: 10.1590/S0004-282X1977000100009, 843241

[7] Evans RJ Benson MK Hughes DT. Abnormal chemoreceptor response to hypoxia in patients with tabes dorsalis. Br Med J. (1971) 1:530–1. doi: 10.1136/bmj.1.5748.530, 5575947 PMC1795314

[8] Fujiki S Fujino M Machida A. Paroxysmal sympathetic hyperactivity caused by neurosyphilis. BMJ Neurol Open. (2024) 6:e000814. doi: 10.1136/bmjno-2024-000814, 39415898 PMC11481103

[9] Task Force of the European Society of Cardiology and the North American Society of Pacing and Electrophysiology. Heart rate variability: standards of measurement, physiological interpretation, and clinical use. Eur Heart J. (1996) 17:354–81. doi: 10.1093/oxfordjournals.eurheartj.a014868 8737210

[10] Fang SC Wu YL Tsai PS. Heart rate variability and risk of all-cause death and cardiovascular events in patients with cardiovascular disease: a meta-analysis of cohort studies. Biol Res Nurs. (2020) 22:45–56. doi: 10.1177/1099800419877442 31558032

[11] Zeid S Buch G Velmeden D Söhne J Schulz A Schuch A . Heart rate variability: reference values and role for clinical profile and mortality in individuals with heart failure. Clin Res Cardiol. (2024) 113:1317–30. doi: 10.1007/s00392-023-02248-7, 37422841 PMC11371886

[12] Jarczok MN Weimer K Braun C Williams DP Thayer JF Gündel HO . Heart rate variability in the prediction of mortality: a systematic review and meta-analysis of healthy and patient populations. Neurosci Biobehav Rev. (2022) 143:104907. doi: 10.1016/j.neubiorev.2022.104907 36243195

[13] Thayer JF Lane RD. Claude Bernard and the heart-brain connection: further elaboration of a model of neurovisceral integration. Neurosci Biobehav Rev. (2009) 33:81–8. doi: 10.1016/j.neubiorev.2008.08.004 18771686

[14] Benichou T Pereira B Mermillod M Tauveron I Pfabigan D Maqdasy S . Heart rate variability in type 2 diabetes mellitus: a systematic review and meta-analysis. PLoS One. (2018) 13:e0195166. doi: 10.1371/journal.pone.0195166, 29608603 PMC5880391

[15] McIntosh RC. A meta-analysis of HIV and heart rate variability in the era of antiretroviral therapy. Clin Auton Res. (2016) 26:287–94. doi: 10.1007/s10286-016-0366-6, 27395409

[16] Heimrich KG Lehmann T Schlattmann P Prell T. Heart rate variability analyses in Parkinson’s disease: a systematic review and meta-analysis. Brain Sci. (2021) 11:959. doi: 10.3390/brainsci11080959, 34439578 PMC8394422

[17] VanderWeele TJ Ding P. Sensitivity analysis in observational research: introducing the E-value. Ann Intern Med. (2017) 167:268–74. doi: 10.7326/M16-2607 28693043

[18] Berntson GG Bigger JT Jr Eckberg DL Grossman P Kaufmann PG Malik M . Heart rate variability: origins, methods, and interpretive caveats. Psychophysiology. (1997) 34:623–48. doi: 10.1111/j.1469-8986.1997.tb02140.x 9401419

[19] Shaffer F Ginsberg JP. An overview of heart rate variability metrics and norms. Front Public Health. (2017) 5:258. doi: 10.3389/fpubh.2017.00258, 29034226 PMC5624990

[20] Goldstein DS Bentho O Park MY Sharabi Y. Low-frequency power of heart rate variability is not a measure of cardiac sympathetic tone but may be a measure of modulation of cardiac autonomic outflows by baroreflexes. Exp Physiol. (2011) 96:1255–61. doi: 10.1113/expphysiol.2010.056259, 21890520 PMC3224799

[21] Billman GE. The LF/HF ratio does not accurately measure cardiac sympatho-vagal balance. Front Physiol. (2013) 4:26. doi: 10.3389/fphys.2013.00026, 23431279 PMC3576706

[22] Eckberg DL. Sympathovagal balance: a critical appraisal. Circulation. (1997) 96:3224–32. doi: 10.1161/01.CIR.96.9.3224 9386196

[23] Miao R Song C Zhang W Ma X Zhang Y Huang Y. A retrospective review of nine cases of atypical neurosyphilis. Front Med (Lausanne). (2025) 12:1603596. doi: 10.3389/fmed.2025.1603596, 40630477 PMC12234453

[24] Mirmosayyeb O Yazdan Panah M Alinejadfard M Oraee S Vaheb S Shaygannejad V. Heart rate variability in people with multiple sclerosis: a systematic review and meta-analysis. Acta Neurol Belg. (2026) 126:67–83. doi: 10.1007/s13760-025-02829-5, 40587005

[25] Studer V Rocchi C Motta C Lauretti B Perugini J Brambilla L . Heart rate variability is differentially altered in multiple sclerosis: implications for acute, worsening and progressive disability. Mult Scler J Exp Transl Clin. (2017) 3:1317. doi: 10.1177/2055217317701317, 28607756 PMC5408506

[26] Byun JI Lee ST Moon J Jung KH Shin JW Sunwoo JS . Cardiac sympathetic dysfunction in anti-NMDA receptor encephalitis. Auton Neurosci. (2015) 193:142–6. doi: 10.1016/j.autneu.2015.08.002, 26275576

[27] Liviero F Scapellato ML Volpin A Battistella M Fabris L Brischigliaro L . Long-term follow-up of heart rate variability in healthcare workers with mild COVID-19. Front Neurol. (2024) 15:1403551. doi: 10.3389/fneur.2024.1403551, 38827576 PMC11141692

[28] Boudreau P Yeh WH Dumont GA Boivin DB. Circadian variation of heart rate variability across sleep stages. Sleep. (2013) 36:1919–28. doi: 10.5665/sleep.3230, 24293767 PMC3825442

[29] Baschieri F Cortelli P. Circadian rhythms of cardiovascular autonomic function: physiology and clinical implications in neurodegenerative diseases. Auton Neurosci. (2019) 217:91–101. doi: 10.1016/j.autneu.2019.01.009, 30744907

[30] Limotai N Somboon T Suanprasert N Rujirussawarawong S Tumnark T Unwanatham N . Exploring cardiac autonomic dysfunction in synucleinopathies with parkinsonism through awake-sleep stages: a possible connection to cardiovascular death. BMJ Neurol Open. (2026) 8:e001412. doi: 10.1136/bmjno-2025-001412, 41573095 PMC12820873

